# Factors Influencing the Use of Mobile Apps and Wearables: Pre- and Post-Surgery Quality of Life Assessment Study

**DOI:** 10.2196/68293

**Published:** 2026-04-21

**Authors:** Laëtitia Gosetto, Emma Cockcroft, Allan Berrocal, Aria Nouri, Ian Porter, Enrico Tessitore, Philippe Bijlenga, Al-Amin Kassam, Jonathan Evans, Jose M Valderas, Katarzyna Wac

**Affiliations:** 1 Quality of Life Lab, University of Geneva, Geneva, Switzerland; 2 Department of Health & Community Sciences, Exeter Collaboration for Academic Primary Care (APEx), University of Exeter, Smeall building, St Luke's Campus, Exeter, EX1 2LU, United Kingdom; 3 Neurosurgery Division, University Hospitals Geneva Medical Center, Geneva, Switzerland; 4 Royal Devon and Exeter Hospital, Exeter, United Kingdom

**Keywords:** quality of life, degenerative cervical myelopathy, liver transplant, total hip replacement, technology-reported outcomes, surgery, wearables, mobile applications, mHealth, smartphone, telehealth, self-management

## Abstract

**Background:**

Quality of life (QoL) is an important surgical outcome, commonly assessed through self-reports, and has the potential to be enhanced by objective information from personal technologies such as smartphone apps and wearables. Understanding patients’ perspectives on this application of personal technologies is scarce.

**Objective:**

This study aimed to identify operational aspects of smartphone- and wearable-based assessments, as well as human and operational factors that may influence the acceptability of already owned (mostly smartphone) or new (mostly wearable) technologies by patients for pre- and post-surgery QoL assessments.

**Methods:**

Through purposive sampling, 41 patients from 3 health care centers in Switzerland, the United States, and the United Kingdom, who were undergoing or scheduled for surgery for degenerative cervical myelopathy (DCM), liver transplantation, or total hip replacement, were interviewed about their perceptions of QoL, current use of smartphones, health apps, and wearables for self-management and their views on using these technologies to assess QoL before and after surgery.

**Results:**

Across the 3 studies (n=41), most (n=36) patients reported improved QoL after surgery, mainly due to reduced pain and fatigue and regained autonomy, while a few patients with DCM reported no change (n=2) or worsening (n=1). Patients were generally comfortable using smartphones and tablets, but few (n=4) used them for health management. Wearables were perceived differently across groups: they were well accepted in transplant@US, moderately in hip@UK, and least in myelopathy@CH. Many patients with DCM found wearables “useless,” believing they added little to their self-awareness or recovery and could not replace human clinical judgment. Others expressed concerns about privacy, complexity, notifications, and battery life, while some acknowledged their motivational value when the data were clearly interpreted. Despite varying acceptance levels, most participants said they would consider using such devices if they contributed to research or improved care.

**Conclusions:**

Given a mostly negative attitude of patients toward wearables, we discuss the use of smartphone-based automated logging of physical functioning (sleep and physical activity) instead. Such logging may be less accurate than a dedicated wearable, but it may be accurate enough to measure their pre- and post-surgery physical functioning changes. Additionally, a smartphone has the advantage of being already well integrated into the daily life of patients from the perspective of its functionality and the patients’ routines, contrary to wearable devices, which would have been provided to the patients in the context of pre- and post-surgery clinical care and require additional attention for their continuous wear, charging, and data synchronization, among others.

## Introduction

Quality of life (QoL) is an important component of surgical outcomes [1-6]. Historically, for example, the American College of Surgeons, among other organizations, called for focusing on “*the end result idea*,” which postulates that it is important to follow up with patients after each operation to determine if it was successful, to learn from negative outcomes, and to develop a method to guarantee future success [7]. This approach was especially appropriate when postoperative morbidity and mortality were very high. The common practice was to observe and use postoperative complications to determine whether the surgery was successful. Nevertheless, given the decline in surgical mortality, a more pronounced focus is put on the morbidity and especially on changes in QoL among patients before and after surgery [8]. The QoL is a complex construct that includes all physical, psychological, social, and environmental domains and encompasses the patient’s general health perception [8].

Currently, patients’ QoL outcomes over a given period (eg, a month) are typically assessed via self-reports leveraging validated patient-reported outcomes (PROs), which are reports on the patient’s health status that come directly from the patient without any interpretation of these responses by a physician or anyone else [9]. PROs are important because the clinical outcomes, which are the primary concern for surgeons, do not always reflect the health aspects that are most important to the patient. The PRO assessments can help the surgeon evaluate whether the patient needs surgery, follow the effect of surgery in the short and long term, and establish priorities for discussions during medical appointments. PROs also allow surgeons to understand and measure the benefits of different procedures from the patient’s point of view. Thus, PROs complement the traditionally measured clinical outcomes by adding the patient’s perspective [10]. However, these assessments are subject to biases affecting self-reporting, including memory and social desirability biases. It is increasingly important, as also emphasized by the regulators, to evaluate the patient’s progress after the surgery with more granularity than self-reports and in ways that are more meaningful for patients than clinical outcomes [2,11]. Such evaluations can inform the assessment of the quality of care and ensure the patient’s safety, for example, influencing the prescription of painkillers [12].

In parallel, personal technologies such as smartphone apps and wearables are becoming increasingly accurate in measuring long-term physical functioning (such as sleep and physical activity), psychological functioning (such as stress), and other outcomes contributing to the patient’s QoL [12]. A recent scoping review has shown that the use of wearable devices can considerably improve clinical outcomes by enabling continuous monitoring. Positive correlations have been observed between PROs and wearable-derived metrics, often referred to as digital biomarkers, suggesting that these objective measures can provide complementary insights into patients’ QoL [13]. Existing evidence supports an association between PROs and digital biomarkers, with moderate correlations reported between observed and predicted PRO values, particularly for domains such as social limitation, frequency and severity of symptoms, and overall QoL. Furthermore, biomarkers linked to activity intensity and volume have been consistently associated with PROs, reinforcing the potential of digital biomarkers to capture meaningful health-related changes beyond self-report [14]. However, the extent to which these technologies can provide complementary, meaningful QoL-related information for patients with surgical conditions, as well as the extent to which these patients would accept these technologies for the QoL outcome assessment, is unknown. It is known that the availability of the technologies does not directly translate into their usage by chronically ill patients [15].

To better understand the core of the patient’s meaningful aspects of QoL, and especially the potential role of personal technologies for assessing these aspects before and after surgery, we selected groups of patients undergoing 3 different surgical procedures performed in 3 different countries (ie, Switzerland, the United States, and the United Kingdom). The diversity of procedures and locations was chosen to provide a broad understanding of the meaningfulness for patients’ QoL-related outcomes that would be possible to be assessed via personal technologies and the patients’ technology acceptance factors regardless of country or a surgery type. The 3 pathologies investigated were degenerative cervical myelopathy (DCM; Switzerland), liver transplant (LT; the United States), and total hip replacement (THR; the United Kingdom). In total, we recruited and interviewed patients who belonged to 1 of the 3 population groups through purposive sampling. The next section reports on the detailed methods leveraged in this study, followed by the results and their discussion.

## Methods

### Overview

This study was reported in accordance with the STROBE (Strengthening the Reporting of Observational Studies in Epidemiology) guidelines [16], and the completed STROBE checklist is provided as Checklist 1.

This overarching study included reanalysis of the datasets collected in 2 qualitative components (Switzerland and the United States) and a patient and public involvement (PPI) workshop (the United Kingdom) [17,18]. The qualitative interviews were primarily guided conversations in which patients provide knowledge, views, and experiences in response to the questions from the researcher [17,19], and all the collected answers were formally analyzed afterward.

The PPI is not designed or conducted as a formal data collection activity. The purpose of the PPI session was to gather broad input from patient representatives on priorities for future research and potential implications for the implementation of digital health tools. No audio or video recordings were made, and participants were not asked to provide personal health information or detailed accounts of their lived experience. Instead, field notes were taken during and after the session to capture general insights and reflections. The PPI allows for more dialogue and 2-way sharing of knowledge, experiences, and perspectives within the research team, developing the research agendas centered around patient experience. Therefore, the PPI notes acquired within this research were used only to contextualize and inform the interpretation of findings and were not included in the formal qualitative analysis or coded as part of the dataset.

This distinction is important in understanding the respective contributions of each method: while interviews generated in-depth, analyzable data on individual experiences and perspectives, the PPI workshop served a consultative and reflective function, aligned with best practices in co-producing future research directions [20].

The differences in methodology for each country are presented in Table 1: referred to as *myelopathy@CH* for DCM in Switzerland, *transplant@US* for LT in the United States, and *hip@UK* for THR in the United Kingdom. The 2 qualitative studies were approved by ethics committees, whereas this was not necessary for the PPI activity. The interview guides are available in the Multimedia Appendix 1.

**Table 1.** Comparison of research methods between the 3 studies.

Study	myelopathy@CH	transplant@US	hip@UK
Year	2021‐2022	2019‐2020	2021
Pathology	Degenerative cervical myelopathy	Liver transplant	Hip replacement
Overall approach	Individual patient–based	Individual patient–based	Group of patients: PPI^a^
Data types	Quantitative (PRO^b^) and qualitative (interview)	Quantitative (PRO) and qualitative (interview)	Qualitative (workshop and survey)
Research methods	PRO: QoL^c^ PROMIS-GH^d^; semistructured interview	PRO: QoL SF-20^e^; semistructured interview: patients and their informal caregivers (‘support persons’)	PPI workshop; semistructured interview; online survey
Devices worn	None	Activity tracker: Fitbit Charge	None
Ethical approval	CCER PB_2018‐00122	Stanford IRB-47833	Not required

a PPI: patient and public involvement.

b PRO: patient-reported outcome.

c QoL: quality of life.

d PROMIS: Patient-Reported Outcomes Measurement Information System-Global Health.

e SF-20: Short Form-20.

Rather than conducting exactly the same study with different cohorts, we have joined forces to meticulously code and analyze what we have already collected across these different studies because it all relates to the same research question. Combining the results from the myelopathy@CH, transplant@US, and hip@UK studies offers a valuable opportunity to compare hypothetical and real-world experiences with digital tools, enriching the overall understanding of patients’ perspectives across different health care, cultural, social, and other important contexts and helping to distill the basic principles that influence human acceptance. Such an approach has been discussed in the literature as promoting the results’ generalization [21,22].

Before combining the study results, the research team acknowledged contextual variability in technology adoption related to cultural and systemic differences across health care settings. In the United States, structural factors such as insurance-based models and an emphasis on individual responsibility may influence digital health adoption [23], whereas European countries with publicly funded systems often promote integration through coordinated policies and national strategies [24], fostering greater trust and engagement [25]. These contextual factors likely shape patients’ willingness to use smartphones and wearables for self-management and QoL assessment before and after surgery [26]. By combining data across these settings, we aimed to identify common factors influencing patients’ engagement with digital tools and their acceptance of such technologies for QoL assessment in diverse health care contexts.

Toward this end, we have combined the results from 3 studies to specifically identify patients’ attitudes toward using passive data collection technologies to assess their QoL. We assumed that such measures can potentially support the existing PROs (especially for the physical functioning and activity) [27,28], and, with a more established and validated passive sensing of wearables functionalities in the future, even replace some PRO-based QoL assessments [29]. The details of each substudy are as follows.

### myelopathy@CH: DCM at the University Hospitals of Geneva (CH)

DCM represents the most common cause of spinal dysfunction in adults [30]. DCM is caused by age-related changes in the spine, including degeneration of the facet joints, discs, and/or vertebral bodies; progressive spinal kyphosis; and ossification, calcification, or thickening of the spinal ligaments. These anatomic changes can lead to progressive spinal cord compression and, consequently, neurological deterioration and a significant decline in QoL [31]. It has been shown that the QoL of patients with DCM is more severely affected than most other chronic diseases in terms of physical functioning, especially physical activity, and is surpassed only by congestive heart failure. This is likewise the case for psychological functioning, behind only back pain and sciatica [32]. Surgical treatment remains the only definitive treatment for DCM and has been shown to improve QoL regardless of the severity of neurological impairment [33].

QoL in patients with DCM has typically been assessed by patient-reported measures similar to those used for other pathologies, including Short-Form Health Survey 36 (SF-36) [34], the Patient-Reported Outcomes Measurement Information System (PROMIS) [35], and the Neck Disability Index [36]. Patients are also assessed by health care practitioner–reported outcomes, such as the modified Japanese Orthopedic Association form [37] or the Nurick Scale [38].

The myelopathy@CH study presented here began with an individual semistructured interview regarding patients’ health status, health self-management strategies, and general use of personal technologies and on their use with regard to personal health management, that is, what they thought of these new technologies, such as smartphone apps and wearables and whether they used them. We would then ask them about their subjective assessment of the QoL (via a semistructured interview) at the time of the interview and retrospectively before surgery (if they were after surgery). Finally, patients completed an online questionnaire for their demographic data and PROMIS Global Health (PRO, PROMIS-GH).

### transplant@US: LT at the Stanford Medical Center (the United States)

LT is the second most frequent transplant after kidney transplant and concerns approximately 9000 individuals yearly in the United States alone. For reference, over 10,000 individuals were on a waiting list for LT as of March 2026 [39]. The quality of these patients’ lives is severely impaired in all areas, especially physical (ie, autonomy in daily living) and psychological functioning, which then influences their overall QoL. According to recent research, the preoperative QoL of these patients improved in all areas of life, especially in physical health, daily activities, sexual functioning, and social functioning, including returning to the workforce [40-42].

There exist several self-report–based QoL assessment methods for patients undergoing LT: the SF-36 [34], Chronic Liver Disease Questionnaire [42], or Karnofsky Performance Status [43]. Owing to the likely cognitive impairment experienced by the patients undergoing transplantation [44,45], especially in the pre-surgery phase, each transplant patient is usually accompanied by an informal caregiver (family member or other acquaintance) for medical appointments. This person is called a “*support person*” by the care team.

The transplant@US study presented here focused on individual semistructured interviews with the same questions as in the myelopathy@CH case described earlier. The interview was conducted with a patient accompanied by a support person. Additionally, it is important to note that the results summarized here originate from a larger study. The overarching transplant@US study implied the involvement of patients and their support persons for 6 consecutive months, with a much larger battery of repeated QoL assessments (including, eg, sleep quality, hope, anxiety, and depression), in addition to weekly short self-assessments (eg, hope, anxiety, and sleep quality). The weekly self-assessments were launched on the patients’ smartphones via the ecological momentary assessment method [46]. The support person’s assessments of the patient’s perceived state (eg, perceived hope, anxiety, and sleep quality) were launched on the support person’s smartphones via a PeerMA method [47]. Additionally, in the overarching transplant@US study, selected patients agreed to wear an activity tracker, the Fitbit Charge, to understand their objective physical functioning patterns, including physical activity and sleep. The results of this study are being reported elsewhere [47].

### hip@UK: THR at Exeter Hospital (the United Kingdom)

THR is one of the most common surgical procedures, with more than 1 million surgeries performed each year worldwide. Due to an aging population, these surgeries are expected to double in the next decade [48,49]. It has been estimated that 93% of surgeries are performed for severe cases of osteoarthritis with pain and physical functioning limitations [49]. Moreover, according to a 2013 meta-analysis, THR brings a real benefit to QoL, especially to physical functioning, within 2 years after surgery [4].

The hip@UK study presented here focused on results from a series of the THR patient involvement workshops. We conducted 3 workshops and online surveys about overall research priorities. The 3 workshops involved 5 patients who would require future hip replacement, 7 patients who had recently had a hip replacement, and 10 patients—a mix of pre- and post-surgery and follow-up meetings with those who had attended either of the previous ones. At the first 2 meetings, the patients discussed important QoL outcomes of their hip surgery, how they imagine these can be assessed beyond self-reports, and their views on using personal smartphones and wearables to measure these outcomes. The final meeting provided feedback and a summary from the initial meeting and discussed the potential burden of QoL outcome assessment and the potential clinical implications of the assessment. Additionally, the patients discussed how they imagine the overall results to affect future research design and planning in the THR. After all the meetings, an online follow-up survey was sent out to gain additional feedback, ask the patients to rank potential QoL outcome measures in order of importance, and gain insight into future research priorities.

### Ethical Considerations

Ethical approval for the myelopathy@CH and transplant@US studies was obtained from the respective institutional ethics committees (CCER PB_2018‐00122 and Stanford IRB-47833). In contrast, the hip@UK study was classified as a PPI project and therefore did not require formal ethics committee approval, as no intervention or collection of identifiable personal data was involved.

For the studies reviewed by ethics committees, participants provided written informed consent using committee-approved consent forms. For the PPI study, consent was implied through voluntary participation, and no additional consent procedures were required. All collected data were deidentified before analysis to protect participants’ privacy and confidentiality. Participants did not receive any compensation for their participation in any of the studies. We confirm that no identifiable images or materials are included in the manuscript or supplementary files.

Participants did not receive any compensation for their participation in any of the studies.

We confirm that no identifiable images or materials are included in the manuscript or supplementary files.

### Data Analysis Approach

In this study, qualitative data were drawn from interviews conducted with participants across the 2 substudies (myelopathy@CH and transplant@US). These interviews constituted the core dataset for the findings presented in this manuscript. These interviews were analyzed first using thematic analysis and, secondly, mapped onto own previous research in this domain.

We conducted a thematic analysis following the Braun and Clarke 6-phase framework [50,51], which provides a systematic yet flexible approach to qualitative data analysis. Researchers first familiarized themselves with the transcripts, then generated and organized initial codes into potential themes based on conceptual patterns. Themes were reviewed, refined, and clearly defined, with illustrative participant quotes used to contextualize findings. Two independent coders reached consensus on the codes and grouped them into thematic clusters.

Second, patients’ negative and positive comments about the personal technologies were coded according to our 2 previous studies [15]: one focusing on factors influencing the quality of experience of mobile apps in the general public [52] and the second discussing these factors in the context of technology use for health self-management by patients with chronic conditions [15].

### Study Participants Details

Patients were recruited at the respective hospitals by the care teams. All patients were informed about the goals of the study, the procedure, the data collected, stored, and processed for the purpose of research, the risks and benefits of participation, and that they could withdraw from the studies and request deletion of their data at any point in time without any negative consequences. Each patient signed the consent form providing the abovementioned information in detail. At the time of recruitment, all patients were assigned an anonymous identification code used throughout the study. The collection of personal information (eg, name and email addresses) was kept to a minimum. Patients’ characteristics are presented in Tables2  3. The details of the 3 studies are further detailed in Table 2.

**Table 2.** Study participants’ details: recruitment channels and participant count.

Study	myelopathy@CH	transplant@US	hip@UK
Recruitment site	Division of Neurosurgery, University Hospitals of Geneva (Geneva, Switzerland)	Clinical Transplantation Unit, Stanford Medical Center (Stanford, CA, USA)	Patient advisory groups (United Kingdom)
Recruitment procedure	Recruited during clinical visits at the neurosurgery department	Identified and contacted through the clinical transplantation service	Recruited via email distribution of flyers through patient advisory networks
Periods of recruitment	May 2021 to February 2022	October 2019 to April 2020	May to July 2021
Inclusion criteria	Diagnosed with DCM^a^; either scheduled for surgery or operated <1 y before assessment	Scheduled for a liver transplant within the next year or had received a transplant <1 y before participation	On a pathway for hip replacement or had recently undergone hip replacement surgery
Exclusion criteria	Operated >1 y before assessment (to minimize recall bias in pre-surgery QoL^b^ assessment)	Transplant performed >1 y before the study	Not applicable (PPI^c^ study)
Number of participants	21	8 (7 support persons) and 6 patients wearing FitBit	12

a DCM: degenerative cervical myelopathy.

b QoL: quality of life.

c PPI: patient and public involvement.

**Table 3.** Study participants details: detailed characteristics of the patients for the 2 qualitative studies.

	myelopathy@CH^a^ (n=21), n	transplant@US^b^ (n=8), n
Age range (y)		
40‐49	3	—^c^
50‐59	7	1
60‐69	5	5
70‐80	4	—
Undefined	2	2
Gender		
Man	12	7
Woman	9	1
Marital status		
Married or in a relationship	14	8
Single	2	—
Other	5	—
Time since surgery		
Before	5	3 (2 transplanted within the study duration)
1‐3 mo	5	3
4‐6 mo	1	2
7 mo to 1 y	10	—

a Quality of life outcomes (at the recruitment): Patient-Reported Outcomes Measurement Information System (score: 0-100, 50 is the population average, higher is better); Global Physical Health Raw Score: mean 61.43 (SD 15.90); Global Mental Health Raw Score: mean 66.90 (SD 17.14; interpretation: above-average physical and mental health in the study population, with a low variability).

b Quality of life outcomes (at the recruitment): Short Form-20 (score: 0-100, higher is better; n=6); physical functioning: mean 52.25 (SD 26.17; moderate, high variability); role functioning: mean 45.83 (SD 51.03; moderate, high variability); social functioning: mean 50 (SD 45.58; average, high variability); mental health: mean 76.67 (SD 11.71; good, low variability); health perception: mean 42.88 (SD 21.50; somewhat negative, high variability); pain: mean 50 (SD 24.49; moderate level, high variability).

c Not applicable.

The QoL instruments used in each cohort target distinct but complementary aspects of health-related QoL. The PROMIS Global Health measure [35], used in the myelopathy cohort, assesses general physical and mental health through 2 subscales: Global Physical Health and Global Mental Health. Higher scores indicate better health status. In the transplant cohort, the Short Form-20 (SF-20) [53] was used. It includes domains such as physical functioning, pain, general health perceptions, and emotional well-being. Like PROMIS, higher scores reflect better functioning.

## Results

### Overview

**Figure 1.**
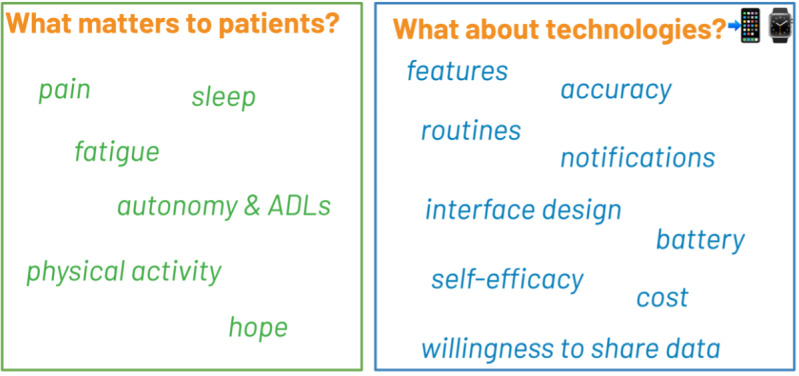
Summary of research findings. ADL: activity of daily living.

In the following sections, we present cumulative results from all 3 studies (N=41). We first describe patients’ perceptions of QoL before and after surgery and relationships between outcomes (eg, pain affecting sleep). We then report on patients’ current use of smartphones, apps, and wearables for health self-management, followed by their attitudes toward using these technologies for pre- and post-surgery QoL assessment. Figure 1 presents the summary of the research findings.

### Patients' Pre-Post QoL Outcomes

Although there were 3 different pathologies requiring surgery, we observed similarities in the factors impacting the patients’ QoL (n=41) and outcomes that have improved or deteriorated following the surgery (n=36). Most patients reported seeing their QoL improve greatly following surgery. A few patients with DCM (n=2) reported seeing no improvement after surgery, although they reported no worsening symptoms either. One patient with DCM did see their condition worsen after surgery.

### Pain

Patients in the DCM and hip replacement groups both reported having pain before surgery (n*=*8). Specifically, patients with DCM had neck pain (n=3) and back pain (n*=*2), while patients undergoing transplantation (n=8) reported abdominal pain. Patients (n=5) have reported that having chronic pain impacts their sleep, which was especially present in the transplant group. One patient with DCM mentioned waking up several times a night because, depending on their position, they had pain or numbness, so they had to wake up to change position (“I wake up at night, stretch my arm and go back to bed.”)*.* In the hip replacement group, both pre- and post-operative patients reported less pain as a key positive impact of surgery. Pain can also impact morale and mental health; as one patient said, “I have pain all the time; it gets on my nerves. I become easily aggressive.”

The surgery clearly relieved pain for the patients with DCM (n=3); following the surgery, they no longer reported the same pain levels they had before the surgery, which improved their mental health as well. A few patients with DCM (n=3, %) still reported some pain, but much less than before the surgery, which did not impact their QoL. A few patients (n=5) reported still having pain despite the surgery. Notably, patients who had a hip replacement mentioned having pain right after the surgery. Patients with DCM (n=9) who had been operated on between 6 months and 1 year before this study also reported sustained pain, especially back and neck pain, as well as hand pain in one case. Such chronic pain can lower morale because it is always present, and patients do not know how to manage it.

### Sleep

Some patients (n=4) reported having difficulty sleeping after the surgery. For hip replacements, patients had to sleep only on their backs, which was uncomfortable. Overall, the improvement in pain after surgery allowed patients to sleep better. Patients (n=7) reported sleeping well or having no problems concerning sleep, and patients who underwent transplantation (n=5) reported better sleep overall. As the latter ones were long-term homebound and not working, they confirmed that they were able to sleep for extended periods in the postoperative period, having naps throughout the day.

### Fatigue

Another symptom frequently mentioned by all the patients before the surgery was fatigue (n*=*18). Patients reported being always tired or very quickly tired, which impacted their activities of daily life and their daily functioning. Patients undergoing transplantation lacked the strength to manage daily activities requiring some force and coordination (eg, shopping). After the surgery, some patients (n=4) reported persistent fatigue. The fatigue experienced by patients who underwent transplantation improved within the first 3 months as their new liver started functioning well.

### Autonomy and Activities of Daily Living

The loss of autonomy is characterized by the inability to accomplish certain activities of daily life without the help of a third person. This loss of autonomy can concern activities of daily living (ADLs), such as feeding, dressing, and toileting, or instrumental ADLs, such as cooking, cleaning, and shopping. Almost all the patients expressed a loss of autonomy during the preoperative phase, as they could no longer be as autonomous as before for several activities. For example, 2 patients from DCM expressed difficulties toileting before the surgery, and they had to ask their caregiver for help with washing. All patients undergoing transplantation (n=8) were additionally confused and unable to function cognitively.

Before surgery, several patients with DCM and patients undergoing transplantation (n=7) reported being unable to stand for long periods, with patients with DCM frequently needing to change position and patients undergoing transplantation fearing falls due to confusion or weakness. Patients with DCM described additional symptoms affecting autonomy, including partial paralysis, loss of strength, tremors, and tingling sensations in the arms or hands, which limited daily activities, such as writing, cooking, or carrying objects weighing more than 5 kg, which requires them to be accompanied to do their food shopping or have them delivered (n=5). Both patients with DCM (n=4) and patients undergoing THR experienced reduced walking ability, often relying on public transport or assistance due to imbalance or fatigue (n=3). Some reported near-falls and a need for accompaniment, describing their walking ability as resembling that of “little old men.”

Some of the patients with DCM reported using a walker to get around. Walking among patients undergoing transplantation was influenced mostly by their fatigue or state of confusion, which prohibited them from even leaving home. In the United States, the walkability of many areas is relatively low, and many patients expressed being practically homebound for most of the preoperative period (n=5).

Patients (n*=*16) also reported being unable to drive before the surgery. A patient with DCM expressed that what handicapped them were his legs, which were “no longer able to press the brake pedal.” Patients undergoing transplantation did not have the cognitive capability and physical force to drive.

After the surgery, a few patients (n=3) still reported having difficulty walking. They tire quickly when walking and can no longer walk long distances: “I have to make a considerable effort to walk 1 km.” One patient also reported having difficulty cleaning themselves. While some need to get assistance, others said they always manage to do it, “but at my own pace.” Following the surgery, patients with DCM (n=3) could also do their household chores worry-free or at their own pace (“It takes me longer to do [the cleaning], but I am autonomous.”). They went back to doing their shopping but always had to be careful not to carry more than 5 kg (n=2). Most patients with DCM were also able to resume their professional activity following their surgery; 3 patients still practice their profession. After surgery, only a few patients with DCM (n=3) mentioned that they still had problems with their arm, such as persistent loss of strength to carry (“things that slip out of your hands*”*), tremors, numbness in their hand, or difficulties writing. “I have a permanently disabled hand; the feeling will never be the same.*”*

Most patients could drive their car as they did before the surgery, especially for patients who underwent LTs (n=11). However, some patients who underwent transplantation were not able and willing to drive in the postsurgery period, as some expressed that they were afraid of being alone and confused in the car (n=3). As they were not working, they expressed that they did not need to start driving rapidly again, and it all started slowly. Despite the surgery, some patients still cannot drive (n=2). For 1 patient with DCM, they could no longer turn their neck to see behind them, so they no longer drove in the city. Another patient could no longer do their job as a taxi driver for the same reason, as the pain while driving handicapped him too much.

### Physical Activity

The major QoL outcome for all the patients discussed for pre- and post-surgery period was physical activity beyond walking. Most patients expressed that they felt impacted by their pathologies because they were not able to do as much physical activity as they did before their diagnosis. Patients (n=3) were happy to be able to resume some physical activities, even if they could not do as much as before. Being able to resume their physical activity was very satisfying for them.

### Hope

Patients awaiting liver transplantation (n=8) expressed that their overall health status depended on their hope of receiving transplants. After transplantation, the improvement in hope was significant—they said they were grateful for the transplant, had high hopes for their own life, and would like to contribute to others’ health and QoL. Some patients expressed that participation in this study was also a way of giving back to the health care system. The hopes for the future were high, although the youngest patients (aged <60 y) were unsure if they could find a job after years out of their profession.

### Personal Technologies Current Use and Attitudes Toward the Future Use

This section discusses results of factors influencing the use of personal technologies such as smartphone apps and wearables by patients in their daily lives, as well as their attitudes for use of these technologies for health self-management and pre- and post-surgery QoL assessments.

### Current Use of Personal Technologies by Patients and ADLs

Most patients with DCM felt comfortable using personal technologies, especially smartphone apps. Eleven patients felt comfortable using smartphones, tablets, and computers. They use their smartphones daily without difficulty; they use them mainly for basic features such as calling, instant messaging systems, internet searches, and photos. The computer, on the other hand, for most patients, was considered a tool for work or administrative tasks (eg, paying bills and accessing bank accounts) and was, therefore, less often used: “impossible to do without (computer) today but as I am still working, I am still confronted with its use.”; “the computer is just for work and at home.” However, they still felt comfortable with this tool. One patient was a computer scientist and expressed feeling particularly comfortable with their computer.

On the other hand, 4 patients with DCM felt moderately comfortable with the smartphone and tablet and were not comfortable with the computer. Here too, patients use their smartphones daily but only for basic features such as calls and instant messaging services but said that they do not dare to do more on their smartphones and prefer asking a relative when they have a problem. “I use my phone a lot. Not very comfortable with it (smartphone), I don’t really know how to install everything.”; “I'm not very comfortable with it, but I know how to use it (new technologies) if I have to. But sometimes I feel like kicking the can down the road.”

Patients who underwent transplantation all own and use a smartphone, and some of them use a tablet and feel comfortable with achieving the needed tasks. Again, it must be noted that their support persons admitted to taking over more complex tasks such as paying bills or scheduling medical appointments due to the patient’s cognitive impairment due to the disease. Hence, the phone and tablet were mostly used for their communication, entertainment, and other personal hedonic needs. A computer was not mentioned at all; just one patient said that they used it to watch movies on a larger screen.

Patients undergoing total hip replacement were comfortable with new technologies, and they mentioned that it was interesting to be able to link in with devices already being used, such as smartphones or smartwatches.

Only one patient with DCM was not comfortable at all with all digital tools. He said they used the smartphone only to call and that their wife used these devices. In addition, 3 patients with DCM who were comfortable with the technologies reported that they could no longer use them because of their condition. One patient used other tools such as a voice-activated system to replace the mouse and send more voice messages. Another patient could no longer use their smartphone or computer because they no longer had sensitivity in their fingers. The third patient mentioned that they could no longer stay more than 30 minutes in front of their computer without having pain in the neck.

### Current Use of Personal Technologies by Patients and Health Self-Management

Although patients with DCM, patients undergoing transplantation, and patients undergoing total hip replacement say they are comfortable with new technologies, very few of them use these for their health management. Only 2 patients with DCM and 2 patients undergoing transplantation had a smartwatch that could measure physical functioning, physical activity, and sleep. Three other patients with DCM had used a connected watch but no longer used it; for 2 patients, the reason was that it had proved “useless,” and for 1 patient, the bracelet had broken, and it was impossible to repair it. Overall, very few patients also used mobile apps for health self-management. Patients undergoing transplantation mostly mentioned accessing apps for their medical appointments and electronic patient records (“myHealth” app). Some patients with DCM (n=4) mentioned looking at their personal statistics taken by the smartphone, such as the number of steps taken during the day. A single patient with DCM who used a smartwatch also used an app for stretching exercises and a meditation app.

As described earlier, we discussed with the patients the factors influencing their use of personal technologies for self-management, which were coded according to our previous studies [15,52]. No new factors were considered beyond those identified by the previous works. The results are as follows.

### Personal Technologies: Features

The majority of patients with DCM evaluated wearable devices as “useless.” For most of them, it was not useful to know the daily number of steps, the quality of sleep, or their heart rate because they knew if they had walked enough or slept well (“I know if I slept well or not. I don’t see the point of the watch.”; “I walk a lot; I don’t need to calculate the number of steps*”*). Most patients with DCM are aware of their day-to-day behaviors and health status and do not find the device appealing. Another point mentioned by patients in all 3 studies was data privacy. They were worried about who could have access to their data and about paying attention to what was being done with their data. Especially, the patients with DCM in Switzerland have expressed that concern.

On a positive note, the patients with DCM and patients who underwent transplantation who had Fitbits (provided to them in the study) have reported looking at the data collected by their smartphone or smartwatch daily. They mainly looked at (from the most to the least mentioned) the number of steps, sleep quality, heart rate, and calories expended. They also thought it was important to have one device that does it all. Patients who underwent transplantation were more open to the device’s features and potential use for self-care. However, patients awaiting surgery were too confused to understand what the numbers meant and how they related to their liver condition. Support persons of patients who underwent transplantation expressed that it would be good for them to know when the patient is sleeping (data from a wearable) and to be able to plan their visit, especially for those not living with the patient but occasionally visiting them.

### Personal Technologies: Accuracy

Patients also questioned the accuracy of these devices. A patient in the hip replacement group said that when they knitted, the device had recorded steps but not when they walked on a treadmill. Another patient reported being perplexed about the accuracy of the data taken for the electrocardiogram. Patients with DCM mentioned that these devices could not replace the actual observations made by physicians. They could not replace humans but rather supplement or notify the presence of red flags but not replace them. On the other hand, patients who underwent transplantation seemed not to question the accuracy as much, likely assuming that it must be accurate enough if the device is provided to them in clinical settings.

### Personal Technologies: User Routine

Patients from the hip replacement group found it positive that the devices were not intrusive in their daily lives, and they could adjust the device to their personal life. Yet, some patients found these devices to be stressful. Having access to all these data daily could make them anxious and hypochondriac. Without medical training, it can be difficult to know what is normal: “I don’t want any of this; measuring his blood pressure, etc is getting paranoid!”; “I’m not interested; maybe one day I’ll get into it. It’s not useful right now. It’s anxiety-provoking to watch your heart rate every day. I trust nature. I don’t need it to know if I’m okay.”; “I leave that (connected watch) to hypochondriacs.”

### Personal Technologies: Notifications

All patients also mentioned that they did not want to be permanently disturbed by devices with notifications. Some patients found that they are already quite dependent on new technologies and already received enough notifications not to have to add more. They would be happy to use these devices if they did not disrupt their day-to-day activities.

### Personal Technologies: Interface Design

Patients with DCM and patients who underwent transplantation were also concerned about the complexity of these wearables. Some patients with DCM and patients who underwent transplantation felt their devices were too complicated for them and feared that they would not know how to configure them. Patients with DCM also found these devices not user-friendly or inviting. Patients who underwent liver transplantation, in particular, as mentioned earlier, overall, may have been too confused to manage the device and its settings.

### Personal Technologies: Battery

One last problem mentioned about these devices was having to remember to charge them regularly. Patients undergoing liver transplantation, in particular, were worried about this.

### Personal Technologies: Self-Efficacy

Patients in the hip replacement group mentioned that device feedback could lower their sense of self-efficacy, that is, the belief that they are able to change, that they are capable. This can upset them, knowing they are in fact walking less than they thought they were. It can also stress them out, fearing that they may perform worse than they imagined. In addition, the pressure could be put on people who will take longer to recover after surgery because of other conditions or the context of their lives. They mentioned that it was very important to be able to customize these devices to each user with goals that will be achievable by the user according to their abilities (eg, adjusting the desired target for the daily number of steps). Conversely, patients across the DCM, transplant, and hip replacement groups reported that postsurgery activity data could enhance motivation, provide reassurance about progress, and support recovery when appropriately interpreted.

### Personal Technologies: Cost

Patients in all 3 studies mentioned the price of these devices as a potential barrier to their acquisition. They may be interested in using them, but they may be too expensive for them, which can create inequalities. On the other hand, patients who underwent transplantation refused to use a “cheap plastic” wearable because they did not want to put it next to their expensive Rolex watch.

### Personal Technologies: Willingness to Share Data

Patients with DCM, patients who underwent liver transplantation, and patients undergoing total hip replacement mentioned that being able to share their data with relatives or physicians was positive. Patients who underwent transplantation especially were hoping that the statistics from a wearable could be shared with a care team, who would then reach out to the patient if abnormalities were seen in the data due to, for example, extended pain periods (especially before surgery).

### Patients’ Attitude Toward Technologies for Measuring the QoL Outcomes

Differences emerged between countries in patients’ perceptions of wearables for QoL assessment before and after surgery. In the transplant@US group, devices were generally well accepted, although some patients required assistance managing them before surgery due to confusion or fatigue. Participants in the hip@UK group were positive overall but expressed some concerns about data sharing and privacy. In contrast, acceptance was lowest in the myelopathy@CH group, where several patients found wearables intrusive or questioned their ethical relevance and ability to meaningfully reflect QoL (eg, distinguishing emotional from physical causes of reduced activity). Nonetheless, most patients indicated they would consider wearing such devices long term if this contributed to research or improved care.

### Comorbidity Challenge

One of the most important results of this study that was especially significant for the patients with DCM, and to some extent for patients undergoing transplantation, was the fact that it is important to measure the patient’s overall QoL due to the presence of symptoms related to comorbidities, which also largely influence their daily life functioning, health status, and QoL. Namely, for myelopathy@CH and transplant@US patients, it is difficult to know what part of the symptoms is due to the pathology of interest or to other related diseases. For example, patients with DCM reported leg problems, but according to neurosurgeons, these problems are often due to a lumbar problem rather than DCM. That specific comorbidity is referred to informally as ‘double hit,’ that is, impacting the neck and the back. Additionally, one patient with DCM and one patient who underwent transplantation mentioned that most of their current poor QoL concerns were not due to myelopathy but due to their cancer-related treatments, including aggressive chemotherapy.

Moreover, for the myelopathy@CH patients, the DCM diagnosis is sometimes delayed, while the QoL is influenced negatively. Patients have reported seeing several specialists over a period of at least a year before seeing the neurosurgeon who diagnosed the DCM and scheduled surgery. Most of the time, they saw specialists who did evoke potential treatments, which, however, improved neither their functioning nor their QoL. Any further implementation of these approaches shall consider these aspects and assess the patients’ QoL holistically.

## Discussion

### Principal Findings

We observed a few similarities between the results of this study with different patients’ subgroups, despite the difference in pathologies, course of diagnosis and treatments, as well as the cultural and economic settings in which the interventions took place. Overall, initially, patients expressed having had a poor QoL before the surgery. What impacted the patients the most in the pre-surgery phase was pain, which, in the long term, affected their morale and their overall mental health. Patients also reported being either constantly tired or becoming tired very quickly. Finally, the other factor that influenced their QoL was the loss of autonomy, with difficulties in walking, standing, driving, shopping, or washing. Another factor mentioned specifically by patients with DCM is the time it takes to diagnose the pathology. Symptoms initially manifest by problems in the hands (eg, tingling, numbness, loss of sensation), and patients are often referred to other specialists. Often, they mentioned wasting up to a year consulting different specialists before finally seeing a neurosurgeon who diagnoses DCM and performs surgery. Indeed, the symptoms of DCM resemble those of other pathologies, such as carpal tunnel syndrome, and additionally, some physicians may hesitate to make more complex neurological diagnoses [54]. This explains why some patients reported having treatments with evoked potential and were diagnosed with DCM later, once their QoL had already deteriorated significantly.

After the surgery, most patients improve their QoL, especially within the first 6 months after surgery. Patients reported having great pain relief, better sleep, gaining autonomy (eg, self-care, driving, or even working), or enjoying physical activities again. But some patients, despite the surgery, still have pain, feel tired, have difficulty sleeping, or cannot drive or walk as much as before. However, for some patients, these persistent negative effects after surgery can be explained by comorbidities, such as a patient undergoing cancer treatment who was weakened by chemotherapy or patients with other spinal conditions that may explain the difficulties encountered in walking and pain.

Most patients with DCM and patients who underwent transplantation were comfortable with the daily use of new technologies, especially with their smartphones that could measure their physical activity and sleep. However, they generally had a poor appreciation of smartwatches, especially the patients with DCM. Most patients thought these devices were considered useless; they did not need them to know if they had slept well or walked enough. The patients also did not want to be bothered by notifications. Patients also feared that the watch would be too complicated. Indeed, the fact that these devices must be easy to use was also noted by patients in 2 other studies [55,56]. Patients have also mentioned that wearables can have a negative effect on self-efficacy if they observed lower results than what they expected (having taken fewer steps than they imagined). Finally, the cost of these devices was also one of the negative points discussed. This contrasts with the results of another study in which patients admitted that cost was not the most important factor in their decision to adopt wearables [55].

However, positive points were also discussed, especially for the transplant@US study. They particularly appreciated that they could view the data collected by the wearables on a daily basis. This also made it possible to see the progress made after surgery, which motivated them to put in more effort. Sharing personal sleep and activity data with relatives or health care practitioners was also positive because it was not considered too intrusive. Patients in a study also found it very useful to be able to share data collected by wearables with health care professionals, with 24.8% of participants using wearables having already shared their data [56]. In our previous studies, we have already observed a significant difference in attitudes between patients in Europe and the United States concerning the sharing of personal data with different stakeholders (either anonymously or not) for various services being provided based on that data [57]. Patients in Europe were generally more cautious when it came to sharing their data. Another possible explanation for the difference in perspectives and evaluations between the patients with DCM and patients undergoing transplantation is the level of their exposure to and familiarity with the wearable devices. Transplant patients had the opportunity to use a wearable device continuously over a 6-month period, allowing them to better understand its functionalities and to develop a more informed, and often more positive, opinion. In contrast, patients with DCM were asked to give their opinion hypothetically, based on the idea of wearing and using such a device, in many cases, without having had any direct or sustained experience with it. This lack of practical engagement may have limited their ability to assess the device’s potential benefits and drawbacks in real-life contexts and could partially explain their more reserved or critical attitudes [27].

From these results, we derive the implications for future research. As we conclude that the factor that most affects the patient’s QoL is pain, it is legitimate to think that wearables should measure pain. This could make it possible to better assess the prioritization of surgery and manage the course of convalescence. However, as such a device does not exist yet, despite huge research efforts [58,59], the only way to measure changes in pain is by measuring changes in functioning and assessing changes in sleep and activity. Specifically, the measurement of the number of steps taken in a day as well as their distribution is interesting because most patients mention difficulty walking before the surgery and an improvement after the surgery. Being able to observe their progress is motivating for them. The change in the number of steps is correlated with physical activity, although this function is not linear and depends on many personal and social factors [58].

Wearable technologies have thus emerged as valuable tools to support such monitoring, especially when linked to mobility and sleep metrics. They provide continuous and passive data that can complement traditional QoL instruments. However, as Wac and Wulfovich [60] point out, these data primarily capture physical function and activity and fall short of encompassing the full range of experiences that matter to patients in their daily lives [60]. Social, emotional, and cognitive dimensions of QoL remain largely inaccessible through wearable sensors alone. For QoL assessment to be truly patient-centered, digital tools must integrate both objective and nonmeasurable, subjective data sources. This may include combining wearable data with ecological momentary assessments, app-based questionnaires, or contextual signals derived from smartphone use and environmental factors. Such multimodal approaches can better reflect the lived experience of patients and yield richer, more meaningful insights into their health trajectories.

Alongside the abovementioned developments, as the field advances toward more patient-centered and real-world assessments of health, it is increasingly important to ensure that emerging metrics, particularly those derived from digital health technologies, are developed in alignment with regulatory expectations. The US Food and Drug Administration has emphasized the need for clinical outcome assessments to be supported by robust evidence demonstrating their validity, reliability, and relevance [61]. In this context, our exploration of wearable-derived data as a complement, or potential alternative, to traditional self-report–based QoL instruments represents a preliminary step toward regulatory and methodological alignment. While traditional self-report–based QoL measures remain valuable, they often lack the sensitivity to capture subtle or short-term changes in a patient’s condition [62,63]. Wearable devices, in contrast, can passively and continuously collect data on key aspects of daily functioning, such as mobility and physical activity, offering a more responsive and granular view of patient health status [64]. Future research should focus on the development and validation of such digital end points within a clear regulatory framework, ensuring that they are both meaningful to patients and appropriate for use in clinical research and practice.

However, in contrast to previous research showing high levels of acceptance for wearables, particularly when they are integrated into clinical care and provide tangible value to patients, our study revealed more cautious attitudes, especially in the United Kingdom and Swiss cohorts. Smuck et al [65] highlight 7 key factors that influence the successful implementation of wearable technologies in health care, including care pathway integration, clinician endorsement, patient-centered design, and clear benefit communication. These structural enablers were largely absent from our European cohorts, where participants discussed wearables hypothetically and outside a care context. In contrast, the US transplant cohort, where patients had direct experience using wearable devices in their clinical care, expressed more positive attitudes. This difference underscores a key aim of our study: to explore how contextual and experiential factors shape patients’ attitudes toward digital health tools. Our findings suggest that acceptability is not only linked to the technology itself but also to its perceived integration, clinical relevance, and user familiarity, reinforcing the need for context-aware implementation strategies.

On the other hand, most patients were reluctant to use wearables, and even if, as patients with DCM indicate, they would be willing to wear them for the QoL outcomes measurement, we would not be able to ensure the quality of the data collected; the patients may forget to charge the device, lose it, or otherwise unintentionally incur data loss [66]. We hypothesize that when designing the technologies for QoL outcome measurement, it is better to favor smartphones and sleep and activity sensors [67]. Indeed, the patients did not want to have to wear a designated smartwatch or other devices on them, but, on the other hand, they all have a smartphone with which they feel comfortable, and which is always with them. Therefore, collecting health data automatically captured by smartphones seems like a good alternative. Indeed, although these data are less accurate than those taken by a wearable, patients will accept the smartphone more.

### Limitations

A key limitation of this study is the use of different study designs and data collection methods across patient groups, which limits comparability across studies due to differences in study design, participant demographics, and contextual factors, which may affect the generalizability of the synthesized findings. However, this methodological diversity also serves as a strength, allowing for the integration of both hypothetical and real-world perspectives on digital tool use. This broader view provides valuable insights into context-specific human factors influencing patients’ acceptance and can guide the development of more flexible, patient-centered digital health solutions.

An important limitation is also that we have great disparity in our samples with low representativeness. That is a result of an overall strategy relying on convenience sampling and linked to difficulty recruiting patients in a short time in a clinical setting during the COVID-19 pandemic; for the patients with DCM and patients undergoing transplantation, the clinical appointments happen only once a week. Additionally, not all patients could express their opinion during the pre-surgery phase. Particularly for myelopathy@CH, for which we have patients who have not yet been operated on (n=5), others who have been operated on for less than 6 months (n=6), and others 1 year after their surgery (n=10).

It would also have been interesting to interview patients before and after their surgery and at several time intervals after the surgery. We could have asked them to complete the same QoL assessment as the SF-36. In this way, we could have observed whether the surgery increased the QoL of the patients and how long after the surgery a better QoL was observed.

There are the following limitations related to the use of the existing PRO-based QoL measures in the patient cohorts. In the myelopathy cohort, the PROMIS Global Health measure, while widely used, lacks condition-specific sensitivity and may not fully capture key symptoms, such as fine motor deficits, subtle functional decline, or pain and fatigue unique to spinal cord impairment. In the LT group, the SF-20, being a shorter and older measure, shows ceiling effects and provides limited insight into emotional well-being, medication burden, or transplant-specific QoL challenges. Both tools, although valuable, fall short in addressing the nuanced and evolving health experiences relevant to each patient population.

Additionally, some results can be explained by recruitment bias, such as the age of the patients undergoing transplantation and related digital literacy, and the cost of wearables, which is perceived as more problematic by people with a low socioeconomic status.

Another limitation occurs in our results related to comorbidity. As some of our data can be explained by comorbidities, it would have been wise to use a questionnaire such as the Charlson Comorbidity Index [68]. This would allow us to consider comorbidities and better control whether the symptoms are better explained by a comorbidity or by the pathology of interest in the study.

### Conclusions and Areas of Future Work

Although the different studies concern different pathologies (eg, myelopathy, hip replacement, and LT), we can observe the same problems that impact the patient’s QoL: pain, loss of autonomy, and difficulties walking. For most patients, their QoL improved after surgery. The patients were pain-free, or their pain was greatly reduced; they regained their autonomy and could do the sports activities they used to do and walk more. Patients were reluctant to using wearables, some finding them unnecessary or not wanting to wear them daily. At the same time, they are very comfortable and use their smartphones daily. Therefore, for a measurement of the change in pre-and post-operative QoL in patients undergoing surgery, we recommend using smartphones instead of wearables to assess mostly the ADLs (including mobility) and sleep, as these are influenced by the level of experienced pain, correlate to a patient’s autonomy, and will reflect the daily level of walking.

For future studies, we should have the same method to allow for better comparison. To have either interviews or focus groups for all the studies and to have the same questionnaires for the patient’s QoL. It is interesting to assess the QoL before and after surgery to observe objectively if there has been an improvement. The use of the EQ-5D or other relevant instruments seems quite appropriate for this purpose but also to get the economic value of improvement in terms of QALYs [69].

## Supplementary material

10.2196/68293 Multimedia Appendix 1 Semistructured interview grid for the studies.

10.2196/68293 Checklist 1 STROBE checklist.

## Abbreviations

ADL: activity of daily living
DCM: degenerative cervical myelopathy
LT: liver transplant
PPI: patient and public involvement
PRO: patient-reported outcome
PROMIS: Patient-Reported Outcomes Measurement Information System
QoL: quality of life
SF-36: Short-Form Health Survey 36
STROBE: Strengthening the Reporting of Observational Studies in Epidemiology
THR: total hip replacement
