# Supplementary material for: Factors Influencing the Use of Mobile Apps and Wearables: Pre- and Post-Surgery Quality of Life Assessment Study
Source: JMIR Form Res. 2026 Apr 21;10:e68293. doi: 10.2196/68293 (PMC13099019; doi:10.2196/68293)
Supplement: Multimedia Appendix 1 [file formative-v10-e68293-s001.pdf]

Semi-directive interview grid for the study of @myelopathyCH

Hello, my name is [name]. I work in collaboration with the doctors in the neurosurgery department. We're working on a project to assess the quality of life of patients with cervical myelopathy and their use of new technologies to help them with their health. So, if you agree, we're going to ask you a few questions about your quality of life and use of new technologies, and then ask you to fill in a very short questionnaire on the iPad.

You don't have to answer every question. If you don't want to answer a question, just let us know. There are no right or wrong answers, so please feel free to answer and be yourself. Your answers will be anonymous, and will only be used for our research.

Semi-directive interview grid

Participant ID: ___________

**How do you use new technologies?**<br>1. For example, smartphone, tablet, computer, connected TV, video game console<br>2. How often? For which tasks?<br>3. What is your overall experience with these technologies?<br>4. Is there anything you're missing in terms of functions/services or other aspects?

**How do you use these technologies for your health?**<br>1. Which ones do you use (connected watch, smartphone, etc.)?<br>a. If smartphone: Which app? Since when?<br>b. If connected watch (or other): which ones? Since when?<br>2. For what purpose? (e.g. to monitor progress towards a goal, e.g. weight, to make a treatment decision, to assist in a discussion with the provider)<br>3. How often?<br>4. What's your experience?<br>5. Is there anything you're missing in terms of functions/services or other aspects?
On a scale from 0 (which is the worst) to 100 (which is the best), how good or bad is your health today? (Feel free to elaborate).

Do you suffer from any other chronic illnesses (e.g. diabetes, cardiovascular disease, COPD)?
Are you independent at home? Outside the home?
How often do you visit your doctor(s)?

How do you feel about your doctor(s) assessing your quality of life? - when they ask you about your pain, sleep and mobility.<br>Are there any gaps in the assessment process, i.e. questions about your quality of life that have never been asked?
Following on from the previous question - if there were gaps, do you think personal technologies could fill them?

We're conducting a research project in which we're imagining the use of personal technologies to monitor the health of patients like you.<br>Would you use your phone/mobile phone for health purposes?<br>And if so, would you be willing to share health data from your phone or wearable device with<br><br>• your doctor?<br>• your family or friends?<br>• Other people (please specify)?

Online questionnaire: PROMIS-GH

Thank you for agreeing to take part in our study!

We're going to ask you to answer a questionnaire about your quality of life and some demographic questions. There are no right or wrong answers, so don't hesitate to have your say!

There are 11 questions in this survey.

ParticipantID: ______________

What's your type?

☐Woman

☐Men

How old are you? __________________

What is your marital status?

☐married / in couple

☐ single

What do you do for a living? ___________________

PROMIS

Please answer each question by checking only one box per line.

	1= Bad	2= Poor	3=Good	4= Very good	5= excellent
Overall, do you think your health is :					
Overall, how would you rate your quality of life?					
Overall, how would you rate your physical health?					
Overall, how would you rate your mental health, including your twin and your ability to think?					
Overall, how would you rate your satisfaction with your activities and your relationships with others?					
Overall, how do you feel you fulfill your usual activities with others and your role in society (whether at home, at work, in your immediate environment, as well as your responsibilities as a parent, child, partner/spouse, employee, friend, etc.)? ...					

	1= Not at all	2= a little	3= Moderate	4= Almost totally	5= totally
How well are you able to perform your daily physical activities, such as walking, climbing stairs, carrying shopping bags or moving a chair?					

Please answer each question by checking only one box per line.

Over the past 7 days...

	1 = Always	2= often	3=sometimes	4= rarely	5= never
How often have you been bothered by emotional problems such as feeling anxious, depressed or irritable?					
	1= very intense	2= intense	3= average	4=light	5=none
How would you rate your average fatigue?					

Over the past 7 days...
How would you rate your average pain?
(0= No pain; 10 = Worst possible pain)

1	2	3	4	5	6	7	8	9	10
☐	☐	☐	☐	☐	☐	☐	☐	☐	☐

Your answers have been recorded.

Thank you for answering our questions.

Semi-directive interview grid for the study of hip@UK

GenEX PPI Meeting 1.1 (pre-op)

Zoom

- **Meeting aim:**
  The first meeting of the GenEX project with pre-op or early arthritic patients. Meeting aimed to inform future research agenda.
  - Important impacts of a hip replacement
  - Views of using phones/wearables to measure and share data with health care provider
  - Barriers to use of phones/wearables to consider

GenEX PPI Meeting 1.2 (post op)

Zoom

- **Meeting aim:**
  The second meeting of the GenEX project with post-op patients, some were on second hip replacement and others had also had revisions. Meeting aimed to inform future research agenda.
  - Important impacts of a hip replacement
  - Measuring recovery after hip replacement.
  - Views of using phones/wearables to measure and share data with health care provider
  - Barriers to use of phones/wearables to consider

GenEX PPI Meeting 2 (Mixed pre and post-op group)

Zoom

- **Meeting aims:**
  1. Feedback from last sessions.
  2. Discuss burden of measurements. How much is too much?
  3. Discuss potential positive and negative impacts of being repeatedly asked about aspects of health which may not be positive (pain, fatigue, limitation) impact of mental health and own assessment of health.
  4. Discussion about design and planning of research (Length of follow up / assessment pre surgery)
  5. What should the research focus be?

Positive & Negative Impacts of being asked about how you are feeling

After meeting online survey (9 responses)

Question 1: What needs researching in the area of hip replacements, patient-reported outcomes and Technology reported outcomes?
